# RNAi-Mediated Knockdown of *Tssk1* and *Tektin1* Genes Impair Male Fertility in *Bactrocera dorsalis*

**DOI:** 10.3390/insects10060164

**Published:** 2019-06-10

**Authors:** Summar Sohail, Kaleem Tariq, Weiwei Zheng, Muhammad Waqar Ali, Wei Peng, Muhammad Fahim Raza, Hongyu Zhang

**Affiliations:** 1 Key Laboratory of Horticultural Plant Biology (MOE), State Key Laboratory of Agricultural Microbiology, China-Australia Joint Research Centre for Horticultural and Urban Pests, Institute of Urban and Horticultural Entomology, College of Plant Science and Technology, Huazhong Agricultural University, Wuhan 430070, Hubei, China; summar.sohail@yahoo.com (S.S.); wwzheng@mail.hzau.edu.cn (W.Z.); waqar3811@gmail.com (M.W.A.); pengwei@cjlu.edu.cn (W.P.); fahim.uca@gmail.com (M.F.R.); 2 Department of Agriculture, Abdul Wali Khan University Mardan, Khyber Pakhtunkhwa 23200, Pakistan; drkaleem@awkum.edu.pk

**Keywords:** RNAi, *Bactrocera dorsalis*, *Tssk1*, *Tektin1*, male fertility

## Abstract

The genetic-based sterile insect technique (SIT) is an effective and environmentally safe strategy to diminish populations of agricultural and horticultural insect pests. Functional characterization of genes related to male fertility can enhance the genetic-based SIT. *Tssk1* has been involved to control male fertility in both mammals and insects. Moreover, *Tektin1* has also been revealed to influence male fertility in both human and mammals. These findings suggested that *Tssk1* and *Tektin1* identified from *Bactrocera dorsalis* could be required for male fertility in *B. dorsalis*. In this study, expression profiles of these two genes were studied at different developmental stages and in various tissues of adult males. Remarkably, it was found that *Tssk1* and *Tektin1* were highly expressed in the testis of mature adult males of *B. dorsalis*. Furthermore, *Tssk1* and *Tektin1* genes were downregulated by using the RNA interference (RNAi) method. Fertility assays including egg laying, hatching, and spermatozoa count were also performed to investigate male fertility of *B. dorsalis*. Results showed that knockdown of *Tssk1* and *Tektin1* caused male sterility up to 58.99% and 64.49%, respectively. As expected, the total numbers of spermatozoa were also significantly reduced by 65.83% and 73.9%, respectively. These results suggested that male sterility was happened wing to the low number of spermatozoa. In conclusion, we demonstrate that *Tssk1* and *Tektin1* are the novel agents that could be used to enhance the genetic-based SIT, or their double-stranded RNA (dsRNA) can be used as biopesticides to control the population of *B. dorsalis*.

## 1. Introduction

The oriental fruit fly, *Bactrocera dorsalis* (Diptera: Tephritidae) is one of the most damaging invasive pest species that attacks more than 250 horticultural crops [1,2,3]. The infestation of *B. dorsalis* has imposed serious losses on the production of fruits and vegetables [4]. Currently, chemical insecticides are being used to control the population of *B. dorsalis* [5,6]. Chemical insecticides have a detrimental impact on human health, wildlife, the environment, and the further development of insecticide-resistant populations [7]. Therefore, there is an urgent need for an alternative control method that can effectively control the insect pest population without using chemical insecticides. An example of such a nonchemical insect control method is the sterile insect technique (SIT).

The SIT is one of the most environmentally friendly insect pest control methods and can be employed to control multiple insect pests. Irradiation-based SIT has been used to control agricultural insect pests, including fruit flies [8,9]. Ionizing radiation sources have been utilized to sterilize the male insects, and these sterile males were afterward introduced into the field [10], although irradiation can potentially exert a negative impact on the fitness of male insects due to which male insects mating competitiveness is reduced. Mating competitiveness of sterile males is required to enable wild males to mate with wild females [10]. Currently, genetic-based SIT is proving very effective at overcoming the pest population without disrupting male insect competitiveness. Functional characterization of more genes related to spermatogenesis of *B. dorsalis* can help to improve the genetic-based SIT. 

Spermatogenesis is a series of molecular and biochemical processes by which spermatogonia undergo a series of proliferations, differentiations, and deformations in the testis, which ultimately forms functional sperm [11,12]. Spermiogenesis is the final stage of spermatogenesis, during which spermatids experience numerous morphological changes, for example, acrosomes formation, nuclear condensation, development of the flagellum, and reorganization of cytoplasm; finally, mature spermatozoa are formed. These significant changes are regulated by gene transcription [13] and protein translation during spermatogenesis. It is recognized that down regulation of spermatogenesis-related genes causes sterility in males of *B. dorsalis* [14].

A variety of protein kinases play critical roles in the regulation of spermatogenesis. One major group of such protein kinases is testis-specific serine/threonine kinases (TSSKs). Until now, five members of the TSSK family have been recognized, and all are significantly expressed in the testis [15]. Different members of the TSSK gene family have been known to be expressed at various stages of spermatogenesis [16,17,18,19]. *Tssk1* was identified as the first member of the TSSKs family [20]. Subsequently, the *Tssk1* gene was used as a probe to clone and describe the molecular characteristics of the *Tssk2* gene in mice [21]. Earlier studies displayed that *Tssk1* was expressed in mature spermatozoa, and many research reports have revealed its multiple roles in mammalian spermatogenesis [22]. Moreover, the expression of *Tssk1* has been identified in the testis of the *Bactrocera tryoni*. Double-stranded RNA (dsRNA) feeding of *Tssk1* in *B. tryoni* significantly silenced the gene and caused male sterility, suggesting its role in the male reproduction of tephritid insects [23]. 

Tektins are another family of proteins that play an important role in the formation of flagella, cilia, and centrioles [24,25,26,27], and stabilize axonemal microtubules. Tektins were initially cloned in sea urchins [25]. Tektins have been identified in various animals, including humans, silk worms, and mice [28,29,30]. Tektins are highly coiled-coiled proteins that constitute filaments in ciliary and flagellar microtubules [31,32]. *Tektin1* is expressed in the human testis and mouse flagella [33]. The evidence for *Tektin1* expression in sperm flagella supports the hypothesis that *Tektin1* plays an important role in the movement of sperm flagella [34]. RNA interference (RNAi), which is a robust and powerful research tool, has been widely used to sterilize males for genetic-based SIT through gene silencing in various insects, including *B. dorsalis* [35,36,37].

Evidence from the previous studies shows that *Tssk1* and *Tektin1* are important genes for male fertility. Transcriptomic analysis from the testis of *B. dorsalis* has revealed that *Tssk1* and *Tektin1* are up-regulated in the testis, but their functions remain unknown [38]. Therefore, we hypothesized that these genes may play important roles in spermatogenesis and can be promising candidates to enhance the genetic-based SIT to control *B. dorsalis* population. We measured the expression profiles and used the RNAi technique combined with fertility bioassays to investigate gene silencing effects on male fertility.

## 2. Materials and Methods 

### 2.1. Flies Rearing

The strains of *B. dorsalis* were reared in the laboratory according to previously described method [39]. Population of adult flies were kept in cages at 28 °C under a 12 h light. The flies were further kept under dark photoperiod for 12 h on artificial diets. The diets consisted of 2.5% yeast extract, 7.5% sugar, 2.5% honey, 0.5% agar, and 87% ddH_2_O. Newly hatched larvae were nourished periodically on banana pulp [40].

### 2.2. Selection of Target Genes for RNAi

Selection of two target genes was made based on previous studies [23,38]. Sequences of *Tssk1* (GenBank accession number: XM_011212727.2) and *Tektin1* (GenBank accession number: XM_011202998.1) were chosen as target genes. Gene-specific primers were prepared for chosen genes using the database of NCBI Primer BLAST (https://www.ncbi.nlm.nih.gov/tools/primer-blast/ (Table 1). Sequence similarity of target genes with other insect species was determined by sequence alignment through ClustalW, and phylogenetic tree was constructed by using Mega 5.1 software. Neighborhood joining method along with 1000 bootstrap was used to construct the tree. 

### 2.3. Expression Profile Analysis

The gene expression was evaluated at all developmental stages, and different body tissues were evaluated through quantitative real-time PCR (qRT-PCR) analysis. For expression analysis at different stages, insects at different developmental stages were collected and washed using DEPC water, including eggs, larva, and pupa. Immature insects were collected 5 h after ecolosion. Adult insects were collected 14 days after emergence. 20 individuals were used for each treatment. For tissue expression analysis, 30 adult insects were dissected under microscope (Olympus SZX12, Olympus, Tokyo, Japan) and immediately collected in DEPC water. Different tissues (head, abdomen, midgut, Malpighian tubules, fat body, and testis) were collected, and total RNA was isolated. Single strand cDNA was prepared by using the cDNA kit (ThermoScientific, USA, Catalog No. k1641). *Tssk1* and *Tektin1* gene expression was analyzed at different developmental stages, and different tissues were analyzed through qRT-PCR.

### 2.4. Preparation of Double Stranded RNAs 

Double stranded RNAs were synthesized using open reading frame (ORF) of *Tssk1* and *Tektin1* of 380 bp and 320 bp fragments. Control EGFP–dsRNA with 495 bp was prepared using the EGFP primers. All dsRNAs of desired genes (*Tssk1* and *Tektin1*) and control dsRNA were prepared using the T7 RiboMAXM Express RNAi System (Promega, Catalog No. P1700). The newly prepared dsRNA was purified by MEGAclear (Ambion) and kept at −80 °C for further use.

### 2.5. Feeding of dsRNA

The newly emerged male and female flies of *B. dorsalis* were employed as experimental materials. Male and females were separated in rearing cages (18 cm × 9 cm × 9 cm). Male insects were starved for 24 h, and then fed 1.0 mL dsRNA of three different concentrations (500 ng/µL, 700 ng/µL, 1000 ng/µL) for 6 h as in our previous study [35]. After 6 h of dsRNA feeding, insects were shifted on normal diet. The dsRNA-egfp was selected as control. 100 insects were used for each treatment. Each treatment set was replicated three times. After 12 days, hatching percentage and sperm count were recorded. 

### 2.6. Quantitative Real-Time PCR (qRT-PCR) Analysis

Impact of various concentrations of dsRNA on gene transcription was investigated using a quantitative real-time PCR. Every 24 h, total RNA was periodically isolated from 10 adult male insects. The commercially available kit (Thermo Fisher Scientific, Waltham, MA, USA) was employed to prepare single strand cDNA. qRT-PCR analysis was carried out on a Bio-Rad iCycler using Universal SYBR Green iTaq™ Supermix (BioRad, Catalog No. 172-5124, Hercules, CA, USA) as per company instructions [41]. Target primers of *Tssk1* and *Tektin1* were prepared for qRT-PCR analysis. Total used 20 μL reaction volume consisted of 0.8 μL of forward and reverse primers; 2 μL cDNA, 6.4 μL ddH_2_O and 10 μL of SYBR Master Mix were used for quantification. The reaction was carried out under optimized thermal cycler amplification conditions for RT-qPCR [14]. The obtained data was examined according to Ali et al. [35].

### 2.7. Reproduction Bioassays

The twenty pairs of dsRNA-treated males and untreated females were separately nourished on a normal artificial diet for 13 days. Afterward, these insects were transferred into a new container for mating. After 24 h, eggs were picked up within 20 min of egg laying from the paper cup placed on banana. The collected eggs were carefully placed and counted manually on A4-sized black paper sheet. Thereafter, hatching efficiency was estimated by placing eggs on banana pulp under controlled environmental conditions. The number of hatched larvae of aged 3–5 days were manually counted. The reproductive capacity of adult males was determined using online calculator compared to those with a control group [42].

### 2.8. Spermatozoa Counts and Sperm Viability Assays

Testis of 12 days old male flies were dissected in a petri dish containing Hayes solution (added mixture of 0.2 g CaCl_2_, 9.0 g NaCl, 0.1 g NaHCO_3_, and 0.2 g KCl in 1000 mL H_2_O). Immediately after being punctured with forceps, 2 μL of flowing sperm was collected. These sperm were diluted with 250 μL of Hayes solution. Sperm numbers were counted with previously described method [43]. In brief, sperm were fixed carefully in ethanol and air dried. Finally, air dried sperm were stained with DAPI for 15 min. Spermatozoa were counted by using a fluorescence microscope. Sperm viability was observed by using the sperm viability kit (Thermo Fisher Scientific, Catalog No. L-7011), which contains two luminous dyes that assisted us to distinguish between dead (red propidium iodide gives red emission) and live sperm (SYBR-14, gives green emission dye) cells. 5 μL of diluted sperm was first incubated with 5 μL of SYBR-14 working solution (2 μL SYBR-14 stock in 98 μL Hayes solution) on a glass microscope slide in the dark room at 25 °C for 10 min, followed by 7 min incubation with propidium iodide. UMNG2 microscope was used (Olympus) to measure sperm viability. Microscope was set at 400 × magnifications, and at least 400 live and dead sperm cells per slide were counted. Dual-stained sperm cells were not included in the data. Sperm viability was obtained for each sample by calculating the percentage of live sperm in the total number of sperms counted. To validate our experimental procedure, we killed the sperm in a sample by placing the sample at −80 °C for 8 h. The viability of all sperm was calculated and observed as stained red.

### 2.9. Data Analyses

Statistical analyses for data of gene expression in various developmental stages and tissues were carried out using statistical software SPSS 19.00 (SPSS. Inc., Chicago, IL, USA). Experimental data of all replicates were analyzed through one-way analyses of variance (ANOVA) using GraphPad prism 5.0 (GraphPad Software, San Diego, CA, USA). For qRT-PCR, an independent Tukey test was performed, and for egg laying and hatching data student *t*-test was performed. The data was normalized as described previously [44].

## 3. Results

### 3.1. Selection of Genes Related to Spermatogenesis

**Figure 1:**
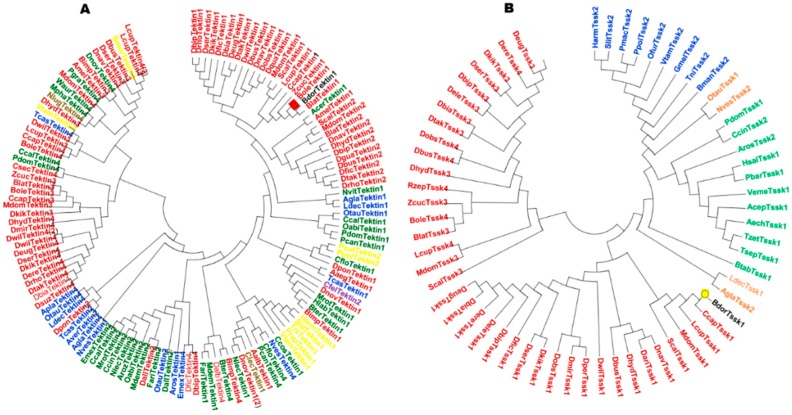
Phylogenetic analysis of Tektin, and Tssk gene family of *B. dorsalis* and different insect species. I41 proteins of Tssk and 63 proteins of Tektin family were analyzed. Different colors indicate different orders of insects. Black color indicates *B. dorsalis* (**A**) mehroon (Diptera), blue (Coloeopltera), yellow (Lepidoptera), green (Hymenoptera), purple (Siphonaptera), and red (Blattodae). (**B**) Mehroon (Diptera), green (Hymenoptera), blue (Lepidopters), and orange (Coleoptera). Names of different insects are Aaeg (*Aedes aegypti*), Acer (*Apis cerana*), Agla (*Anoplophora glabripennis*), Amel (*Apis mellifera*), Apla (*Agrilus planipennis*), Aroz (*Athalia rosae*), Aver (*Asbolus verrucosus*), Bdor (*Bactrocera dorsalis*), Bimp (*Bombus impatiens*), Blat (*Bactrocera latifrons*), Bole (*Bactrocera olea*), Bter (*Bombus terrestris*), Bmor (*Bombyx mori*), Ccal (*Ceratina calcarata*), Ccap (*Ceratitis capitata*), Ccin (*Cephus cinctus*), Ccos (*Cyphomyrmex costatus*), Cfel (*Ctenocephalides felis*), Cflo (*Camponotus floridanus*) Clec (*Cimex lectularius*), Csec (*Cryptotermes secundus*), Dall (*Diachasma alloeum*), Dbip (*Drosophila bipectinata*), Dbus (*Drosophila busckii*), Dele (*Drosophila elegans*), Dere (*Drosophila erecta*), Deug (*Drosophila eugracilis*), Dfic (*Drosophila ficusphila*), Dgua (*Drosophila guanche*), Dhyd (*Drosophila hydei*), Dkik (*Drosophila kikkawai*), Dmir (*Drosophila miranda*), Dnav (*Drosophila navojoa*), Dobs (*Drosophila obscura*), Dqua (*Drosophila guanche*), Dnov (*Dufourea novaeangliae*), Drho (*Drosophila rhopaloa*), Dser (*Drosophila serrata*), Dsuz (*Drosophila suzukii*, Dtak (*Drosophila takahashii*), Dwil (*Drosophila willistoni*), Dobs (*Drosophila obscura*), Dpon (*Dendroctonus ponderosae*), Dser (*Drosophila serrata*), Dnav (*Drosophila navojoa*), Dalb (*Aedes albopictus*), Deug (*Drosophila eugracilis*), Dbia (*Drosophila biarmipes*), Emex (*Eufriesea mexicana*), Fari (*Fopius arisanus*), Harm (*Helicoverpa armigera*), Hlab (*Habropoda laboriosa*), Lcup (*Lucilia cuprina*), Mdem (*Microplitis demolitor*), Ldec (*Leptinotarsa decemlineata*), Mdom (*Musca domestica*), Mrot (*Megachile rotundata*), Mpha (*Monomorium pharaonis*), Nlec (*Neodiprion lecontei*), Nvit (*Nasonia vitripennis*), Nlug (*Nilaparvata lugens*), Nves (*Nicrophorus vespilloides*), Oabi (*Orussus abietinus*), Otau (*Onthophagus taurus*) Prap (*Pieris rapae*), Pcan (*Polistes canadensis*), Pdom (*Polistes dominula*), Pgra (*Pseudomyrmex gracilis*), Scal (*Stomoxys calcitrans*) Slit (*Spodoptera litura*), Tcas (*Tribolium castaneum*), Vtam (*Vanessa tameamea*), Tni (*Trichoplusia ni*), Waur (*Wasmannia auropunctata*), and Zcuc (*Zeugodacus cucurbitae*). Neighborhood joining method along with 1000 bootstraps was used to construct the tree.

We have selected two genes, *Tssk1* (XM_011212727.2) and *Tektin1* (XM_011202998.1), which are related to male fertility based on previous studies [23,33]. Partial nucleotide sequences of *Tssk1* and *Tektin1* were 1761 and 1607 bp long. ORF of these two genes encodes amino acids of 900 and 1266bp, respectively. Nucleotide sequence of *Tssk1* gene shares more than 95% identity with *B. tryoni* [23]. Amino acid sequence of *Tssk1* also aligned with other insects (Figure S1). The alignment results indicate that *Tssk1* of *B. dorsalis* shares 70–95% identity with other insects, whereas the sequence alignment of *Tektin1* sequence with the *Tektin* genes of other insects shows identity 60–95% with other insects (Figure S2). The sequence analysis through multiple alignments suggests that these are naturally conserved genes. Phylogenetic analysis of Tektin gene family shows that *Tektin1* gene of *B. dorsalis* is closely related to *Tektin1* gene of *Bactrocera Latifrons* (Figure 1A), whereas phylogenetic analysis of *Tssk* protein family indicates that *Tssk1* of *B. dorsalis* is closely located to *Tssk1* gene of *Cerattis Capitata* (Figure 1B).

### 3.2. Expression Profiles of Target Genes 

**Figure 2:**
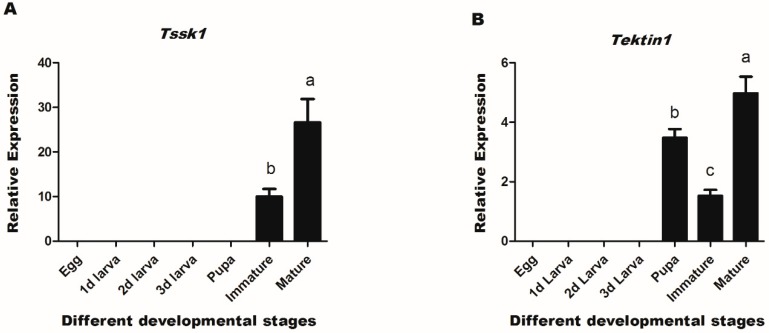
Expression profiles of *Tssk1* and *Tektin1* at different developmental stages of *B. dorsalis* male insects. (**A**) Expression profiles of *Tssk1* at different developmental stages. (**B**) Expression profiles of *Tektin1* at different developmental stages. Different letters above the bars indicate significant differences (least significant difference) in one-way analysis of variance (ANOVA) (*p* < 0.05).

Previously transcriptomic analysis [38] of cDNA library identified testis-specific genes, of which two genes (*Tssk1* and *Tektin1*) were upregulated in mature male adults. To verify the putative mRNA expression pattern of these two genes (*Tssk1* and *Tektin1*) in the sexual maturation of male adults, expression profiles of target genes *Tssk1* & *Tektin1* were measured through qRT-PCR at different developmental stages of male insects. Results demonstrated that *Tssk1* mRNA was expressed in immature and mature adults, whereas *Tektin1* gene was detected in pupae, immature, and mature adults (Figure 2A,B). Both genes were more highly expressed in mature adults than immature adults.

**Figure 3:**
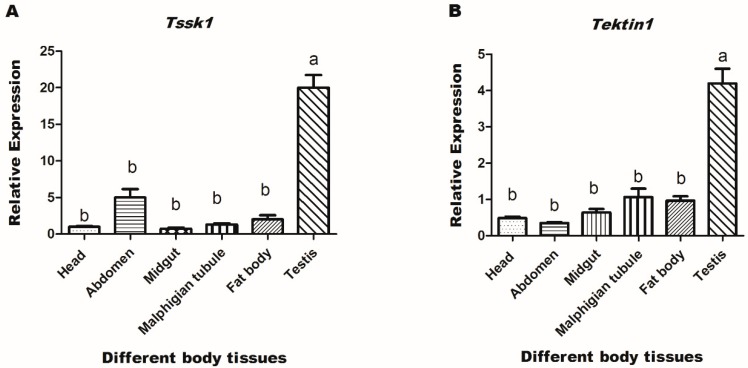
Expression profiles of two genes in different body tissues of *B. dorsalis* males. (**A**) Expression profiles of *Tssk1* in different body tissues. (**B**) Expression profiles of *Tektin1* in different body tissues. Different letters above the bars indicate significant differences (least significant difference) in one-way ANOVA at *p* < 0.05.

Expression profiles of *Tssk1* and *Tektin1* were also investigated in different body tissues of the male adults of *B. dorsalis*. qRT-PCR results indicated that *Tssk1* and *Tektin1* were expressed in all body tissues, but their expression was much higher in testis than in other tissues (Figure 3A,B).

### 3.3. Effect of dsRNA Feeding on Tssk1 and Tektin1 Expression 

**Figure 4:**
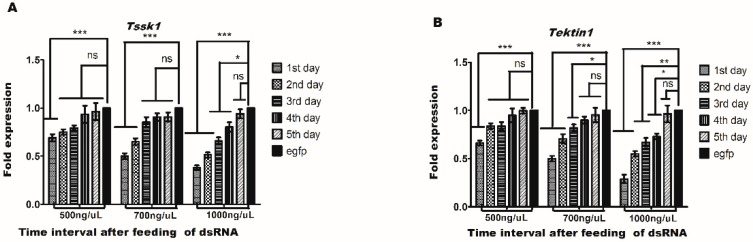
Gene silencing of *Tssk1* and *Tektin1* gene in males of *B. dorsalis* caused by oral feeding of different concentrations of their dsRNAs. The control was treated with ds-egfp. (**A**) Fold change in *Tssk1* gene transcription. (**B**) Fold change in *Tektin1* gene transcription. One-way ANOVA was used to analyze the results (*p* < 0.0001, Tukey test).

The highest expression of *Tssk1* and *Tektin1* in the testis of male adults indicated their important functions in male fertility. Therefore, knockdown experiments using RNAi were carried out to investigate the functions of these two genes (*Tssk1* and *Tektin1*) in the male fertility of *B. dorsalis*. Real-time PCR (qRT-PCR) analysis was performed after oral feeding of dsRNA of *Tssk1* and *Tektin1* genes for 6 h on newly emerged male adults. The results showed successful delivery of dsRNA, as evidenced by the fold change in the gene expression. Fold change in the gene expression was measured for five consecutive days. The results showed that highest down-regulation effect was found in the 1st day with the feeding of *dsTssk1* at concentrations of 500 ng/μL, 700 ng/ΜL, and 1000 ng/μL, which was about 31%, 50%, and 62%, respectively (Figure 4A). With the feeding of *dsTektin1* at concentrations of 500 ng/μL, 700 ng/ μL, and 1000 ng/μL, maximum downregulation in the gene transcription was also found on the first day, at approximately 33%, 50%, and 71%, respectively (Figure 4B). 

**Figure 5:**
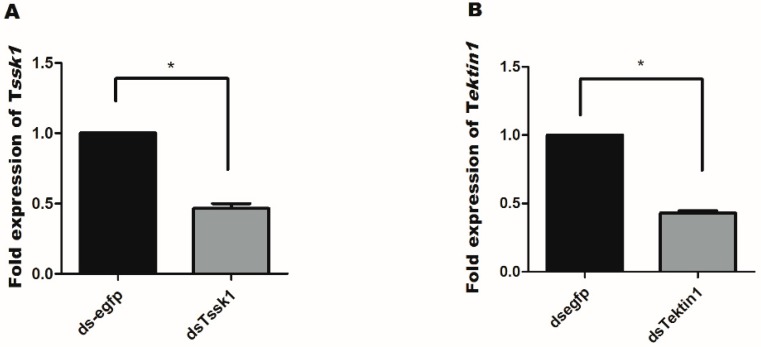
Gene silencing of *Tssk1* and *Tektin1* in testis of males of *B. dorsalis* caused by oral feeding of dsRNA at the concentration of 1000 ng/uL. The control was treated with ds-egfp. (**A**) Fold change in *Tssk1* gene transcription. (**B**) Fold change in *Tektin1* gene transcription. One-way ANOVA was used to analyze the results. * shows significant difference at *p* < 0.05.

Further, mRNA level of *Tssk1* and *Tektin1* was measured in testis after 24 h of dsRNA feeding. Reduction in mRNA level was also observed in testis with dsRNA feeding of both genes (Figure 5A,B).

### 3.4. Effect of Genes Silencing on the Reproductive Capacity of Males

**Figure 6:**
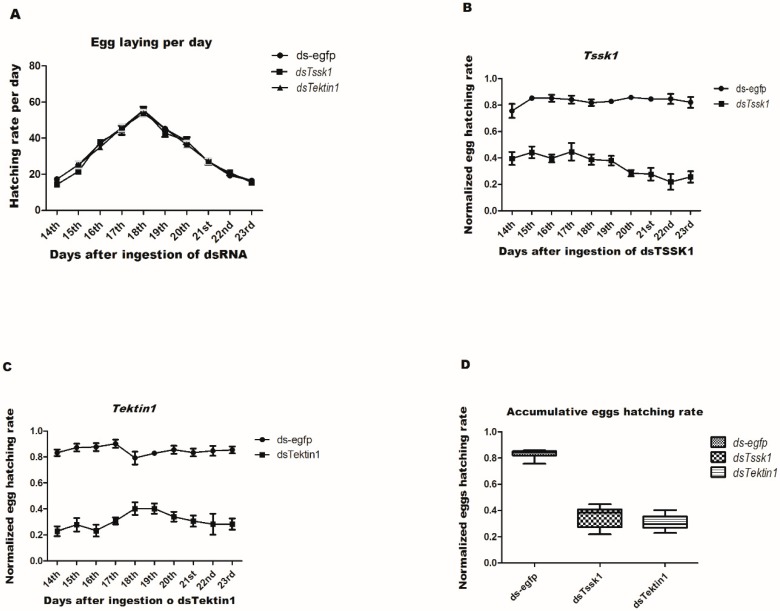
Reduced expression of *Tssk1* and *Tektin1* in testis have decreased the male fertility in *B. dorsalis*. (**A**) Average number of eggs laid per female per day after crossing with males treated with *dsTssk1*, *dsTektin,* and control (ds-egfp) group. (**B**) Average number of eggs hatched per day obtained from females crossed with *dsTssk1*-treated males, compared to control (ds-egfp) group. (**C**) Average number of eggs hatched per day obtained from females crossed with *dsTektin1* treated males, compared to control (ds-egfp). (**D**) Accumulative eggs hatching rate. One-way ANOVA along with student T-test was used to analyze the effects of treatments (*p* < 0.05).

To investigate the effects of gene silencing of *Tssk1* and *Tektin1* on the reproductive capacity of male flies of *B. dorsalis* and daily numbers of eggs laid, hatching assays were carried out. Results indicated that there was no significant difference in the numbers of eggs laid between dsRNA feeding of target genes and control (ds-egfp) group (Figure 6A). The same genes (*Tssk1* and *Tektin1*) were chosen, and the effect of the dsRNA feeding on male fertility was analyzed. The target genes exhibited significant impact on the hatching rate of eggs. Sterility in *Tssk1* and *Tektin1* knockdown males was observed up to 58.99% and 64.49%, respectively, compared to the control (ds-egfp) group (Figure 6B,C).

### 3.5. Effects of Gene Silencing on the Quantity and Quality of Spermatozoa

**Figure 7:**
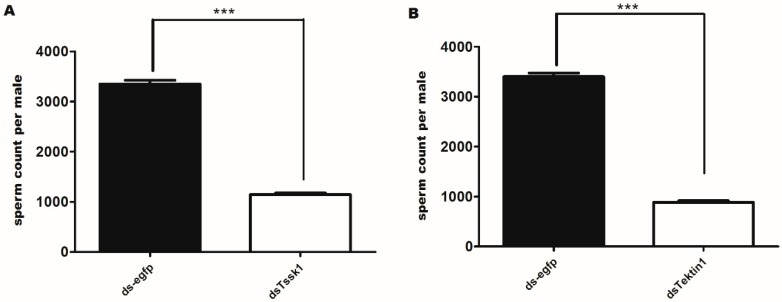
Reduced expressions of *Tssk1* and *Tektin1* in testis have affected the average number of spermatozoa. (**A**) Average number of spermatozoa in males treated with *dsTssk1*, compared to control (ds-egfp) group. (**B**) Average number of spermatozoa in males treated with *dsTektin1*, compared to control (ds-egfp) group. The data were analyzed using T-test. *** indicates *p* < 0.001.

**Figure 8:**
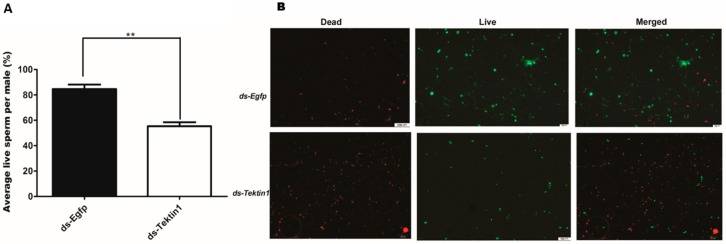
Reduced number of live sperms in the *Tektin1* knockdown males (**A**) Percentage of live sperm significantly decreased in flies fed with *dsTektin*, compared to the control (ds-egfp) group. (**B**) Represents the percentage of live spermatozoa per male. Red indicates dead sperms; green indicates live sperms. One-way ANOVA, along with student t-test, was used to analyze the results. ** indicates *p* < 0.01.

To explore the reason for male sterility, we further measured the number of spermatozoa in the testis of 14 days old males treated with *dsTssk1*, *dsTektin1*, and ds-egfp (control) group. Significant decrease in the numbers of spermatozoa was noticed in *dsTssk1-* and *dsTektin1*-treated males, comprising 65.83% and 73.9%, respectively, compared to control (Figure 7A,B). It is not confirmed whether decrease in the number of sperms is the main reason for sterility in males (64.49%). We assumed that there are multiple reasons for reduced fertility. Thus, the viability of sperm in the treated and control flies was examined. Interestingly, more dead sperm were found than live sperm in the *Tektin1*-treated males than control males. Total live sperm in treated flies were reduced up to 55% compared to control group (Figure 8A,B). These results suggested that disruption of *Tssk1* and *Tektin1* genes affects male fertility by affecting the production of mature spermatozoa and their viability.

## 4. Discussion

Sterile insect technique (SIT) is an environmentally friendly pest control approach that has already been employed in *B. dorsalis* [45]. Up till now, successful implementation of SIT at commercial level has been very critical to creating a vast number of high-quality sterile males. In the present study, *Tssk1* and *Tektin1* were knocked down using RNAi to enhance the genetic-based SIT for the management of *B. dorsalis*. Firstly, we detected the expression profiles of *Tssk1* and *Tektin1* at different developmental stages and in different tissues of the males of *B. dorsalis*. Highest expression of both genes was observed in testis of mature male adults of *B. dorsalis*. These results were consistent with the studies indicating that *Tssk1* and *Tektin1* are essential for male fertility [23,29]. We may suggest that these genes have important functions in spermatogenesis of target insect *B. dorsalis*. To our knowledge, we have identified for the first time the mRNA expression of two genes (*Tssk1* and *Tektin1*) throughout the life span of male of *B. dorsalis* and studied their silencing effects on reproductive capacity of male flies.

Further detailed study on the expression pattern of *Tssk1* and *Tektin1* in different tissues of *B. dorsalis* revealed that both genes were expressed in all tested tissues. It is suggested that *Tssk1* and *Tektin1* might be involved in several physiological processes and regulate the protein stability in insects [22,26,32,33]. During sexual maturation, important changes happen at the transcriptional level in insects [46]. In the present study, both genes exhibited different expression patterns between immature and mature male adults. This kind of different expression pattern suggests that they may play multiple roles in the reproductive development of male adults of *B. dorsalis*. 

RNAi in our current study have reduced the efficacy of target genes, which is consistent with the previous studies of RNAi in *B. dorsalis* [3]. In contrast, RNAi in some lepidopteran insects proved ineffective [47], which might be due to inappropriate dsRNA concentration or due to environmental effects. We used the dsRNA feeding method because it was more convenient than microinjection. It is evident from the previous study that feeding of dsRNA is effective for gene functional study or gene screening on a large scale [48]. Nucleotide sequence selection, appropriate concentration, and suitable application methods are prerequisites to attain the careful silencing effects of dsRNA. Considerably long-lasting effects of *Tssk1* and *Tektin1* deficiency were examined on male fertility of *B. dorsalis,* which ultimately resulted in reduced egg hatching rate compared to their controls (Figure 5). Males of *B. dorsalis* typically take two weeks to reach sexual maturity [38,49], which provided us with sufficient time to deliver enough dsRNA to impact the maturing male’s reproductive capacity. To our surprise, when insects were fed with dsRNA of target genes before sexual maturation, it resulted in a decreased egg hatching rate.

The present results demonstrated that dsRNA application of *Tssk1* gene reduced mRNA expression and reproductive capacity in male flies of *B. dorsalis* (Figure 5B). These results are consistent with previous studies that showed that dsRNA of *Tssk1* in *Bactrocera tryoni* silenced the gene effectively and caused male sterility [23]. In insect spermatogenesis, a *Tssk1* protein is involved in post-meiotic chromatin remodeling, which is encoded by *Tssk1* gene [38]. Mutation or knockdown of *Tssk1* gene caused male sterility in mice, drosophila, and Queensland fruit fly [22,50]. In our *Tssk1* knock-down experiments, the impaired male fertility of *B. dorsalis* might be due to the defects in post-meiotic chromatin remodeling. *Tektin* is a microtubule specific protein identified in different animals including silkworm and is indispensable for sperm motility [29]. Knock-down of *Tektin1* gene impaired male fertility of *B. dorsalis* due to decreased number of spermatozoa and their viability. The defects in sperm motility could be the possible cause of decrease in number of spermatozoa.

## 5. Conclusions

The present study reveals that *Tssk1* and *Tektin1* play important roles in male fertility of *B. dorsalis*. Our results help illuminate the molecular mechanism regulating spermatogenesis in males. Interestingly, feeding dsRNA of *Tssk1* and *Tektin1* genes significantly downregulated the expression of *Tssk1* and *Tektin1* in males of *B. dorsalis*. The disrupted expression of *Tssk1* and *Tektin1* affected the reproductive capacity significantly, resulting in a reduced number of spermatozoa. The reduced quantity and quality of spermatozoa ultimately resulted in male sterility. It is believed that *Tssk1* and *Tektin1* may be promising targets for the development of genetic-based SIT, or that their dsRNA could potentially be used to eliminate the population of *B. dorsalis*.

## Figures and Tables

**Table 1:** Primer used for gene expression analysis and RNA interference (RNAi).

**Primer Name**	**Primer Sequences for qRT-PCR**
*Tssk1*-F	CTCCAATCGCCAACTGAATA
*TSSK1*-R	ATTTGTGTACGAAATCCGAG
*Tektin1*-F	TGTGGATGAAACCAAAGACA
*Tektin1*-R	CCAACGATTTCTCCTTCAGA
*16S-F*	CTCGTCCAACCGTTCATACC
*16S-R*	CTGACCTGCCCACTGAAGTT
**Primer Name**	**Primer Sequence for dsRNA Synthesis**
*dstssk1*-F	GGATCCTAATACGACTCACTATAGGATCACCCAAACATCATACAGA
*dstssk1-R*	GGATCCTAATACGACTCACTATAGGCAATTTCGGATCGTATGGC
*dstektin1*-F	GGATCCTAATACGACTCACTATAGGAGACATGCAAAATCAAACGG
*dstektin1-R*	GGATCCTAATACGACTCACTATAGGCGTGTAGCAAATAGCGTAAC
*dsGFP-F*	GGATCCTAATACGACTCACTATAGGATACGGCGTGCAGTGCT
*dsGFP-R*	GGATCCTAATACGACTCACTATAGGATGATCGCGCTTCTCG

## Supplementary Materials

The following are available online at https://www.mdpi.com/2075-4450/10/6/164/s1, Figure S1: Sequence analysis of *Tssk1*. Multiple alignment of *Tssk1* proteins from *B. dorsalis* and 15 other insect species were performed using clustalW method. Mega 5.1 software was used. Blue, orange, red, and green indicate 70%, 80%, 90%, and 100% sequence similarity, Figure S2: Sequence analysis of *Tektin1*. Multiple alignment of *Tektin1* proteins from *B. dorsalis* and 14 other insect species were performed using ClustalW method. Mega 5.1 software was used. Blue, orange, red, and green indicate 60%, 80%, 90%, and 100% sequence similarity.
