# Supplementary material for: RNAi-Mediated Knockdown of Tssk1 and Tektin1 Genes Impair Male Fertility in Bactrocera dorsalis
Source: Insects. 2019 Jun 10;10(6):164. doi: 10.3390/insects10060164 (PMC6627857; doi:10.3390/insects10060164)
Supplement: Supplementary file 1 [file insects-10-00164-s001.pdf]

RNAi-Mediated knockdown of *Tssk1* and *Tektin1* genes impair male fertility of *Bactrocera dorsalis*

Summar Sohail[1], Kaleem Tariq[2], Weiwei Zheng[1], Muhammad Waqar Ali[1], Wei Peng[1], Muhammad Fahim Raza[1] and Hongyu Zhang[1,*]

[1] Key Laboratory of Horticultural Plant Biology (MOE), State Key Laboratory of Agricultural Microbiology, China-Australia Joint Research Centre for Horticultural and Urban Pests, Institute of Urban and Horticultural Entomology, College of Plant Science and Technology, Huazhong Agricultural University, Wuhan 430070, Hubei, People's Republic of China; summar.sohail@yahoo.com

[2] Department of Agriculture, Abdul Wali Khan University Mardan, Khyber Pakhtunkhwa, Pakistan; drkaleem@awkum.edu.pk

* Correspondence: hongyu.zhang@mail.hzau.edu.cn (H.Z.)

Supplementary Data

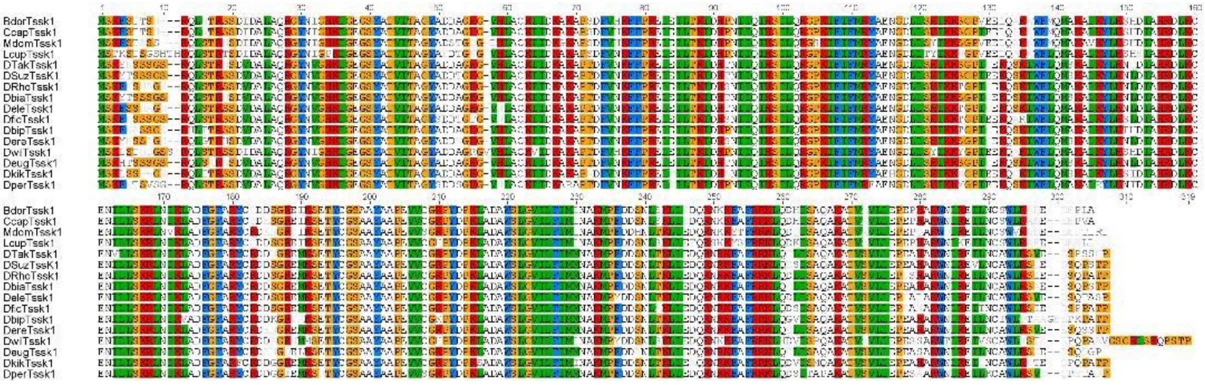

Figure S1. Sequence analysis of *Tssk1*. Multiple alignment of *Tssk1* proteins from *B. dorsalis* and 15 other insect species were performed using ClustalW method. Mega 5.1 software was used. Blue, orange, red and green colors indicate 70%, 80%, 90% and 100% sequence similarity.

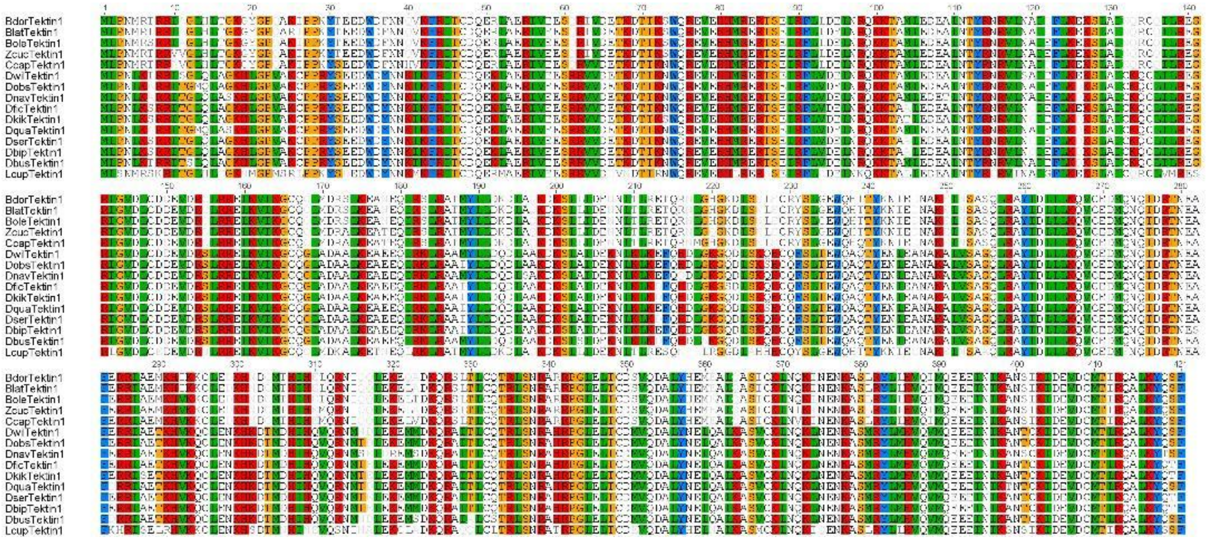

Figure S2. Sequence analysis of *Tektin1*. Multiple alignment of *Tektin1* proteins from *B. dorsalis* and 14 other insect species were performed using ClustalW method. Mega 5.1 software was used. Blue, orange, red and green colors indicate 60%, 80%, 90% and 100% sequence similarity.
